# *Burkholderia contaminans* Biofilm Regulating Operon and Its Distribution in Bacterial Genomes

**DOI:** 10.1155/2016/6560534

**Published:** 2016-12-14

**Authors:** Olga L. Voronina, Marina S. Kunda, Natalia N. Ryzhova, Ekaterina I. Aksenova, Andrey N. Semenov, Yulia M. Romanova, Alexandr L. Gintsburg

**Affiliations:** ^1^N.F. Gamaleya Federal Research Center for Epidemiology and Microbiology, Ministry of Health of Russia, Gamaleya Street 18, Moscow 123098, Russia; ^2^I.M. Sechenov First Moscow State Medical University, Moscow 119991, Russia

## Abstract

Biofilm formation by* Burkholderia* spp. is a principal cause of lung chronic infections in cystic fibrosis patients. A “lacking biofilm production” (LBP) strain* B. contaminans* GIMC4587:Bct370-19 has been obtained by insertion modification of clinical strain with plasposon mutagenesis. It has an interrupted transcriptional response regulator (RR) gene. The focus of our investigation was a two-component signal transduction system determination, including this RR.* B. contaminans* clinical and LBP strains were analyzed by whole genome sequencing and bioinformatics resources. A four-component operon (BiofilmReg) has a key role in biofilm formation. The relative location (i.e., by being separated by another gene) of RR and histidine kinase genes is unique in BiofilmReg. Orthologs were found in other members of the Burkholderiales order. Phylogenetic analysis of strains containing BiofilmReg operons demonstrated evidence for earlier inheritance of a three-component operon. During further evolution one lineage acquired a fourth gene, whereas others lost the third component of the operon. Mutations in sensor domains have created biodiversity which is advantageous for adaptation to various ecological niches. Different species* Burkholderia* and* Achromobacter* strains all demonstrated similar BiofilmReg operon structure. Therefore, there may be an opportunity to develop a common drug which is effective for treating all these causative agents.

## 1. Introduction

The* Burkholderia cepacia* complex (*Bcc*) bacteria are opportunistic pathogens which cause nosocomial infections and are especially dangerous for cystic fibrosis (CF) patients. Analysis of* Bcc* strain diversity in Russian healthcare units and in CF patients demonstrated 5 species:* B. cenocepacia*,* B. multivorans*,* B. stabilis*,* B. contaminans*, and* B. vietnamiensis* [1]. Among these,* B. cenocepacia* was more abundant, and the Russian epidemic strain ST (sequence type) 709 belonged to this species. However,* B. contaminans* ST102 was also isolated from CF and non-CF patients [1]. Moreover, this strain is known to have an intercontinental spread across the world [2].

Presently,* Bcc* eradication is complex and, in most cases, impossible, which leads to chronic infections in the lungs of CF patients. Biofilm formation is the principal reason for bacterial stability in CF patients' respiratory tracts.

The history of biofilm observation is long. But, in spite of studies of planktonic and aggregated forms of microbes that occurred hand in hand, the importance of the biofilm phenomena for medicine was first postulated by Hoiby and Axelsen only at the beginning of 1970s, based on observations of CF patients with chronic* Pseudomonas aeruginosa* lung infection [3]. Later, the first biofilm conference, in 1996, yielded better understanding of the significance of biofilm infection in medicine and marked the beginning of intensive microbial biofilm research. Many different approaches were used: conventional light microscopy; electron microscopy and confocal laser scanning microscopy for biofilm architecture and composition investigation; direct and accidental mutation of biofilm-forming strains; transcriptomic analysis; and differential measurement of biochemical pathway activity and metabolite concentrations of planktonic and sessile cells [4]. Hence we now know that a large number of genes are involved in so complicated process of biofilm formation. For instance, comparison of high- and low-biofilm producing* B. pseudomallei* strains revealed 563 differentially regulated genes [5]. It should be noted that upregulated genes related to two-component signal transduction systems and a denitrification enzyme pathway [5].

A surprising result came from the work of Romanova et al. [6] on nondirectional insertion mutagenesis of high biofilm producer (HBP) clinical strain* B. contaminans* GIMC4509:Bct370, when just one of 1000 plasposon insertions had lost the ability to form biofilm. This LBP strain named* B. contaminans* GIMC4587:Bct370-19 had only one interrupted gene. DNA sequencing of a fragment adjacent to the insertion site identified it as the transcriptional regulator gene* ompR*, which is the part of the two-component signal transduction system (shortly two-component system, TCS). The TCS array consists of a protein histidine kinase (HK) and a response regulator (RR) protein. It is now known that TCSs mediate several different bacterial processes: chemotaxis, aerobic/anaerobic regulation, sporulation, and differentiation [7], as well as biofilm response [8].

The purpose of our investigation was the determination of this key in biofilm formation TCS, part of which is found transcriptional regulator. Detailed study of the BiofilmReg operon structure and evolution could have significant medical applications.

## 2. Materials and Methods

### 2.1. Bacterial Strains and Their Origins

All strains used came from the Gamaleya Institute Microbial Collection (GIMC): high biofilm producer (HBP) clinical strain* B. contaminans* GIMC4509:Bct370 (ST102, PubMLST id 1264) and lacking biofilm production (LBP)* B. contaminans* strain GIMC4587:Bct370-19. The LBP strain was obtained by insertion modification of clinical strain with plasposon pTnMod-RKm by Romanova et al. [6].

### 2.2. DNA Isolation and Genomics

Preparation of genomic DNA for the whole genome sequencing was performed as described [9]. Whole genome sequencing of* B. contaminans* strains was performed according to the manufacturer's (Roche) guidelines for the next generation sequencing (NGS). Two protocols were used for shotgun-sequencing library preparation: rapid library and pair-end library.

### 2.3. Data Acquisition and Processing

DNA sequence assembly into scaffolds was performed with 454 Sequencing System Software v.2.7 and v.3.0 (Roche). To aid in assembling individual chromosome we used data from reference strains:* B. lata* strain* Burkholderia* sp. 383;* B. contaminans* strain MS14;* B. ubonensis* strain MSMB22;* B. cenocepacia* strains: J2315, DDS 22E-1;* B. cepacia* strains: DDS 7H-2, ATCC 25416. The software Rapid Annotations Subsystems Technology and SEED [10, 11] were used for annotating the genome of* B. contaminans* strains. BioProjects PRJNA349796 and PRJNA349797 were registered in GenBank with BioSample Accessions SAMN05933033 for GIMC4509:Bct370 and SAMN05933042 for GIMC4587:Bct370-19. Now the genomes are in the process of the chromosomes assembling.

### 2.4. Bioinformatic Analyses

Complementary protein description, prediction of domains, signal peptides, and protein cellular localization have been performed by NCBI BLAST [12], InterPro server [13, 14], TMHMM Server v. 2.0 [15], SignalP 4.1 Server [16], and PSORTb version 3.0.2. [17]. Promoter sequence prediction has been performed by BPROM (Prediction of Bacterial Promoters) [18, 19] and NNPP (Neural Network Promoter Prediction) [20] servers. Operon borders have been predicted with help of operon predictor: PTools04a (BioCyc Database Collection) [21]. Searches of the NCBI database for orthologs of operon components and gene was performed with the aid of the KEGG ORTHOLOGY (KO) Database [22, 23] and Biocyc Database [24]. Multiple alignments of nucleotide sequences were created in a MEGA 6.0 [25] environment using Multiple Sequence Alignment tools [26]. The numbers of nucleotide differences per site were counted as pairwise distances. Percent similarity and divergence coefficients have been determined by MegAlign 5.05 [25]. Amino acid sequence analysis tools in these same software packages were also used. Searches using NCBI BLAST have been performed for identification of significant sequences, containing sites of phosphorylation, intermolecular recognition, polypeptide, and DNA binding.

### 2.5. Phylogenetic Analysis

Phylogenetic analyses of polypeptide sequence data were performed in MEGA 6.0 [25]. Evolutionary history was inferred by using the Maximum Likelihood (ML) method based on the JTT matrix-based model [27]. The percentage of trees in which the associated taxa clustered together is shown next to the branches. Initial tree for the heuristic search was obtained automatically by applying Neighbor-Join and BioNJ algorithms to a matrix of pairwise distances estimated using a JTT model and then selecting the topology with superior log likelihood value. Trees were drawn to scale, with branch lengths measured as the number of substitutions per site. All positions containing gaps and missing data were eliminated.

Phylogenetic analysis of* 16S rDNA* nucleotide sequences was carried out in MEGA 6.0 [25]. The evolutionary history was inferred by using ML method based on the general time reversible model GTR+G [28], which was chosen as an optimal evolution distance model derived from Modeltest based on the Akaike information criterion [29]. Initial tree for the heuristic search was obtained by applying the Neighbor-Joining method to a matrix of pairwise distances estimated using the Maximum Composite Likelihood (MCL) approach. A discrete Gamma distribution was used to model evolutionary rate differences among sites (6 categories (+G, parameter = 0.3082)). Bootstrap analyses were performed with 500 replicates.

## 3. Results and Discussion

### 3.1. Localization of Biofilm-Switch Response Regulator (RR) in* B. contaminans* Genomes

The clinical HBP strain* B. contaminans* GIMC4509:Bct370 and its modification, LBP strain* B. contaminans* GIMC4587:Bct370-19, were the objects of whole genome sequencing (WGS). Assembling the genomes in scaffolds allowed us to suggest a candidate position for the interruption position of the plasposon and then to find the neighbor genes of RR. Upstream of the insert site and on the same sense there were three genes with own promoters. The distance between the nearest outside gene and RR was 269 bp. This intergenic region included predicted promoter region, a transcription start site, and a 5′ untranslated region (UTR). The promoter region was located at 164–213 bp upstream RR gene start codon, according to NNPP server. The positions of consensus −10 box and −35 box were detected at −192 bp and −212 bp, respectively, with help of BPROM server. The 5′ UTR was surprisingly long for Prokaryotes: 173 bp. The suggestion of our prediction we found in Sass et al.'s experimental work [30]. Authors analyzed the RNA extracted from* B. cenocepacia* J2315 biofilm and revealed 187 CDS (coding sequence), which featured long 5′ UTR of >150 nt, for transcriptional regulators, nucleotide binding, and membrane proteins. Among these 187 CDS was BCAL1443 (two-component regulatory system, response regulator protein) orthologous to our RR.

**Figure 1:**
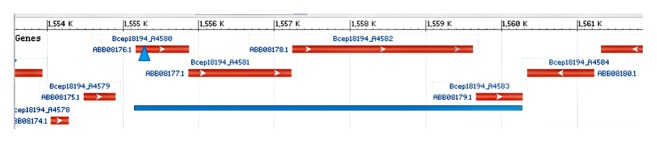
BiofilmReg operon location on Burkholderia sp.383 chromosome 1 (GenBank: CP000151.1). A4580: 1555167…1555871, two-component transcriptional regulator, winged helix family; A4581: 1555868…1557229, peptidoglycan-binding LysM; A4582: 1557240…1559627, periplasmic sensor signal transduction histidine kinase; and A4583: 1559665…1560279, hypothetical protein (http://www.ncbi.nlm.nih.gov/). Blue triangle is in the position of the gene interruption by plasposon. Blue line marks the genes of operon.

The three same sense downstream genes are genes of peptidoglycan-binding protein (PBP) with additional FecR domain, histidine kinase (HK), and uncharacterized protein (UnP) of DUF4136 superfamily (Figure 1). The next open reading frame (ORF) has been located on the antisense strand. The start codon of PBP gene was inside the RR coding region. The distance between the PBP and HK genes was 10 bp and 27 bp between HK and UnP genes. There was not any promoter downstream RR gene all the way to the first gene on the antisense strand according to promoter sequence prediction by BPROM and NNPP servers. So, basing our conclusion on the distances between the four adjacent genes in the same DNA strand and on the availability of a single transcriptional promoter we predict that their organization reflects a common transcriptional operon. Since the RR interruption by the plasposon had switched off the strain biofilm formation entirely, we had named the operon “Biofilm Regulating” (shortly, BiofilmReg). The intact operon of HBP strain has been submitted to the NCBI GenBank database, with Accession Number KP288492. The LBP strain operon sequences have Accession Numbers KP288491 and KU252679.

As mentioned earlier, RR and HK are usually assumed to be a cognate pair, because they lie next to one another within the same operon [31]. In contrast, here the PBP gene is embedded between the RR and HK genes, so they are not “genomic neighbors” in Sheng et al.'s [32] interpretation, because the distance between them is more than 300 bp. Because some researchers who work on bioinformatic analysis of TCS in whole genomes might doubt our evidence that the HK gene from BiofilmReg operon is part of an operon with RR, we have done further investigations to show that these genes form natural functional units within a single operon.

### 3.2. Diversity of Two-Component Transcriptional Regulator (TCTR) Genes in* B. lata* Genome

Is this case of a coregulated gene inserted between RR and HK unique? To answer this question we searched for all two-component transcriptional regulator (TCTR) genes in the reference* B. lata* genome. We found 37 TCTR genes: 49% on chromosome 1 (3.69 Mb, NC_007510.1) and nearly equal numbers, 27 versus 24%, respectively, on chromosome 2 (3.59 Mb, INSDC NC_007511.1) and chromosome 3 (1.4 Mb, INSDC NC_007509.1) even though the second chromosome is twice as big as the third. Eleven TCTRs were found to be encoded by a single gene; the remaining 26 TCTRs were organized in operons. Among these 26, 22 operons were two-component types in accordance with the evidence that the average bacterial operon size is 2.2 genes [33]. The remaining four operons included more than two genes. The biggest contained eight genes whose products participated in phosphate transport. Another one was a three-component type located on the second chromosome. The last two operons had four-components. One of them included genes of the DUF4390 family uncharacterized protein and rRNA SAM-dependent methyltransferase as well as the RR and HK. The second one was the BiofilmReg operon. For the majority of these operons the gene adjacent to TCTR was HK. Only in the BiofilmReg operon was a PBP gene embedded between the RR and HK genes. Since gene organization in an operon is a means to coordinate expression functions [34] we next attempted to understand the possible functions of proteins encoded by BiofilmReg analyzing there domains.

### 3.3. Analysis of Proteins Domains in BiofilmReg Operon Components

First we analyzed the domains of the TCTR (Table S1, in Supplementary Material available online at http://dx.doi.org/10.1155/2016/6560534) in reference* B. lata* genome. According to the NCBI BLAST results, two conservative domains are present in TCTR: receiver and DNA binding. Together they form a multidomain polypeptide, having a Pfam classification [35]. Most of the TCTRs examined, about 70%, had a winged helix-turn-helix (wHTH) DNA binding domain (PF00486), 19% were the representatives of the LuxR family (PF00196), 5% were simple HTH_8 (PF02954) structures, and the last 5% belonged to the HTH-AraC (PF00165) family (Table S2). The most abundant group was subdivided into eight subgroups according to their multidomain characteristics. One of the common types, the CreB family, includes RR from the BiofilmReg operon; CreB is carbon source responsive response regulator that belongs to the CreBC two-component system. Investigation of this system in* E. coli* has demonstrated its participation in controlling genes involved in acetate [36] and ribose metabolism [37], in the maltose regulon [38], and in the pentose phosphate pathway [39] and genes which repair DNA damage associated with the replication fork [40]. Avison et al. [41] have named CreBC “the heart of metabolic regulation” in* E. coli* [41]. RR has localized in cytoplasm of bacterial cell.

The next component in the BiofilmReg operon that we examined is the gene for an uncharacterized conserved protein containing LysM and FecR domains. This is named according to COG4254 (clusters of orthologous groups) [42]. The InterPro resource classified this protein as an uncharacterized conserved protein UCP02964, LysM, PA4035. Orthologs of this gene were variously named: uncharacterized protein (UniProtKB U2H3R6), peptidase M23B (A0A0J6M8Q8), peptidoglycan-binding LysM (Q39H89), and FecR family protein (A0A088U8M6). The structure of LysM domain is known, and a function in peptidoglycan binding is predicted for it. It is found in a variety of enzymes involved in bacterial cell wall degradation [12]. The second domain is FecR, which is involved in regulation of iron dicitrate transport and is probably a sensor that recognizes iron dicitrate in the periplasm [12]. The InterPro service predicted for the protein product of the second component of BiofilmReg a signal peptide and the main part of the protein localized outside of the cytoplasm, which agrees with putative function for this domain.

Third gene in BiofilmReg operon is a gene for a histidine kinase or periplasmic sensor signal transductor histidine kinase. This HK is a multidomain protein. The first domain starting from the N-terminus is transmembrane; the second is a CHASE2 domain (pfam05226), which is an extracellular sensory domain. Environmental factors that are recognized by CHASE2 domains are not known at this time [12]. The next three HK structural elements are transmembrane domains. The subsequent PAS domain is a second sensor domain, which is not present in all HK types [43]. This adaptable domain can monitor changes in light, redox potential, oxygen, or small ligands, depending on their associated cofactor [7]. PAS domain is localized in cytoplasm. The next two domains have the same localization. These are (1) dimerization and phosphotransfer and (2) catalytic and ATP-binding. All together these last domains form multidomain. According to COG classification (COG3852) the HK from BiofilmReg operon is nitrogen specific, having multidomain NtrB [12].

The fourth and final component of the operon is the gene for an uncharacterized protein with a DUF4136 domain. This domain has been found in bacterial lipoproteins [12]. According to InterPro this polypeptide has a signal peptide and the main part of the protein has external localization.

### 3.4. A Model of BiofilmReg Protein Localization

**Figure 2:**
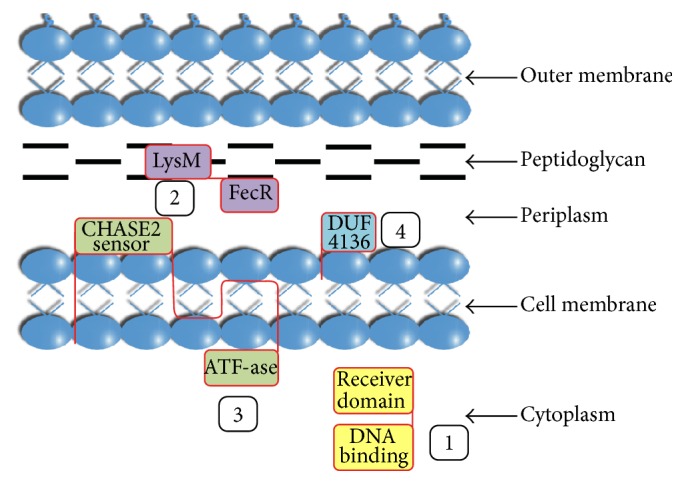
Components of BiofilmReg operon topology in bacterial cell predicted by InterPro Service (http://www.ebi.ac.uk/interpro/). The components are as follows: (1) two-component transcriptional regulator; (2) uncharacterized conserved protein, containing LysM and FecR domains; (3) periplasmic sensor signal transduction histidine kinase; and (4) uncharacterized DUF4136 superfamily protein.

In summary, we present a proposal for colocalization of the four described proteins in bacterial cells (Figure 2).

The periplasm contains two proteins and the signaling domain of HK. One is binding to the rigid exoskeleton (peptidoglycan), which determines cell shape [44], the second is bound to the lipids of the outer or inner membranes. The PBP and HK units sense different signals, which can be transmitted to RR in the cytoplasm and alter target gene expression.

The interruption of this operon by plasposon pTnMod-RKm insertion destroyed all four genes' transcription. Only short fragment of RR gene (148 bp) rests before plasposon sequence. Promoter, detected by BPROM and NNPP servers in the end of plasposon, is divided by the second part of RR gene (564 bp) from the next ORF and so cannot be active. As a result Romanova et al. detected the absence of biofilm formation in vitro by modified strain [6].

### 3.5. Searching of Orthologs of BiofilmReg Operon Components

Do such operons occur frequently in other known bacteria? We searched for orthologs of BiofilmReg operon components to answer this question. First we analyzed Gram-negative bacteria of classes Beta- and Gammaproteobacteria, which are often recorded among opportunistic microorganisms that cause nosocomial infections. A cohort of 45 genomes belonging to 21 genera was examined. A result was considered positive if at least two adjacent components of the operon were detected together in the panel of genomes we searched (Table 1).

Among Gammaproteobacteria, only two* Pseudomonas* strains,* P. aeruginosa* PAO1 and* P. fluorescens* PCL1751, had a couple of orthologous genes. These appear to be an exception. In the class Betaproteobacteria, only the Burkholderiales order had genera containing orthologs of the BiofilmReg operon genes. Various species of the* Burkholderia* genus included fully sized operons in their genomes (Tables 1 and 2). However, other genera in the Burkholderiaceae family had individual species with orthologs of the operon.

Thus,* Pandoraea thiooxydans* contained a couple of the target genes (Table 2), but there were no orthologous genes in* P. pnomenusa* (CP006900.2). Three genera of Alcaligenaceae and a single species,* Ralstonia pickettii*, from the Ralstoniaceae family had orthologs of operon genes. Representatives of two other families of the order Burkholderiales—Comamonadaceae and Sutterellaceae—had no genomes with orthologs. Overall 21 genomes with orthologous genes for BiofilmReg operon have been identified and analyzed (Table 2). Among them there is the Russian epidemic strain* Achromobacter ruhlandii* ST36 (GenBank Accession Number CP017433.1) [45], whose operon is submitted in GenBank with Accession Number KU252680. Almost all* Burkholderia* genomes have an identical operon structure: RR, PBP, HK, and UnP. However, in* L. mirabilis* (*Burkholderiaceae*) and* A. faecalis* (*Alcaligenaceae*), genome UnP was substituted by glutamyl-tRNA reductase and AraC family transcriptional regulator, respectively. Representatives of* Achromobacter, Bordetella,* and* Ralstonia* genera had three-component operons without UnP. Finally, the operon of* P. thiooxydans* DSM 25325 was the most divergent in Burkholderiales order and included only two genes: PBP and HK.

In one gammaproteobacterium a related operon was found in the* P. aeruginosa* PAO1 genome. This consisted of just PBP with a truncated LysM domain and an intact FecR domain, plus HK. Despite the alteration in PBP it was classified by NCBI BLAST analysis as COG4254 too [12].

Given the interesting distribution of these operons we now asked: What is their evolutionary history? To reconstruct the original operon structure in their common ancestral bacterium we analyzed the phylogeny of the listed microorganisms with help of* 16S rDNA* gene sequences as a base of biosystematics [46].

### 3.6. Phylogeny of Burkholderiales Representatives and* P. aeruginosa* Based on* 16S rDNA* Gene Sequences

**Figure 3:**
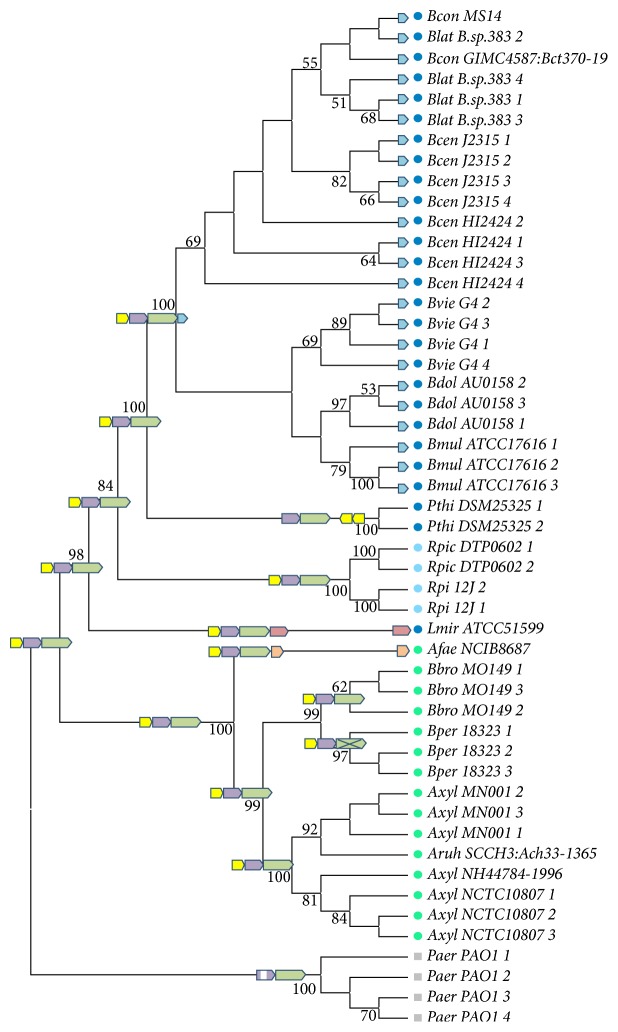
ML phylogenetic tree of Burkholderiaceae representatives and* P. aeruginosa* based on* 16S rDNA* gene sequences. Dark blue: Burkholderiaceae, blue: Ralstoniaceae, green: Alcaligenaceae, and grey:* Pseudomonas aeruginosa.* Operons schematic representation is given on the branch nodes according to Table 1 symbols.

A Maximum Likelihood* 16S rDNA* gene tree (Figure 3) has been created for 50 sequences, which included all identified* 16S rDNA* gene copies of 21 representatives of the families Burkholderiaceae, Alcaligenaceae, Ralstoniaceae, and Pseudomonadaceae (Table 2). It should be noted that* 16S rDNA* gene copies of some genomes have differences in the sequence, so the number of* 16S rDNA* gene copies increases in more than two times the number of genomes in the analysis.

The phylogenetic tree revealed two main clusters of the Burkholderiales representatives, corresponding to the Alcaligenaceae and Burkholderiaceae families. Representatives of Ralstoniaceae were embedded inside the Burkholderiaceae cluster as a distinct group. The Alcaligenaceae family cluster (Bootstrap Index, BI, 100%) included* Achromobacter, Bordetella,* and* Alcaligenes* species, and the Burkholderiaceae cluster (BI 98%) included* Burkholderia*,* Pandoraea, Lautropia,* and* Ralstonia* species. It should be noted that representatives of each genus formed separate clades inside the two major clusters. The* P. aeruginosa* PAO1 operon is the most divergent 2-component operon with an altered PBP and a normal HK. In contrast to* P. aeruginosa* PAO1 (Pseudomonadaceae, Gammaproteobacteria), which is treated as the outgroup, all Burkholderiales genomes contain orthologous operons with at least three components: RR, HK, and PBP being thus consistent with them having a common origin. The three-component operon structure was observed in representatives of two families Ralstoniaceae (*R. pickettii*) and Alcaligenaceae (*A. xylosoxidans, A. ruhlandii, A. insuavis, B. bronchiseptica,* and* B. pertussis*). And four-component operon was detected predominantly in representatives of Burkholderiaceae (*B. contaminans, B. lata, B. dolosa, B. multivorans, B. vietnamiensis, B. cenocepacia,* and* L. mirabilis*) and only in one* A. faecalis* strain of Alcaligenaceae. So it is clear that the three-component operon (RR, PBP, and HK) represents the ancestral state for the major clusters.

It is interesting to trace the evolution of whole operon and its components across the different taxa. For example, in the genome* Bordetella pertussis* 18323 the damaged HK gene cannot code catalytic domain and remains present in the operon only as a pseudogene. In genus* Ralstonia* we detected operon only in some* R. pickettii* strains.

This species differs in chromosome number from others in the genus* Ralstonia*. Here,* R. solanacearum* has only one chromosome,* R. mannitolilytica* has two, and* R. pickettii* has three chromosomes, as do most representatives of the Burkholderia genus which are dangerous for CF patients and for patients of the department of reanimation and intensive therapy as nosocomial infection. One strain,* R. pickettii* 12J, had a conventional version of the three-component operon (Table 2) located on chromosome I. However, in the genome of* R. pickettii* DTP0602, three-component operon, has been found on chromosome II, indicating the possible translocation of a full-sized operon. The PBP structure of this strain has provided additional support for this suggestion. The PDP sequence had an additional fragment at its C-terminal end, which was identified as COG4733, phage-related protein, tail component [12].

In the* P. thiooxydans* DSM 25325 genome, the two operon genes (PBP and HK) lie together on the same sense strand as usual, but the orthologous RR gene is duplicated 3′ and set in the reverse direction on the complementary DNA strand. This arrangement suggests complex recombination and translocation events.

The* Burkholderia* species,* L. mirabilis* (Burkholderiaceae), and* A. faecalis* (Alcaligenaceae) have probably each independently gained an extra (fourth) operon component during their evolution. In contrast to the general similarity of RR, HK, and PBP between these operons, the extra component of the* A. faecalis* operon is an AraC family transcriptional regulator, while the fourth component of the* L. mirabilis* operon is a glutamyl-tRNA reductase [12]. The extra component of all other* Burkholderia* operons belongs to the DUF4136 superfamily, whose function is still unknown. The various functions of the fourth components may indicate that these operons have been recruited in different metabolic pathways, probably involving different signal perception and transduction functions depending on bacterial lifestyle. The presence of these components in operons from different phylogenetic lineages among Burkholderiaceae and Alcaligenaceae representatives also supports the view that these were gained by them rather than lost from all other phylogenetic lineages.

If the conventional version of the three-component operon has an ancient origin, then we can ask: How much genetic distinction has accumulated in operon genes during evolution along different phylogenetic lineages? To answer this question we analyzed the polypeptides encoded by a selection of these operons.

### 3.7. Analysis of the Sequence Diversity in Individual Operon Components

Our first proteins comparison was done at the level of domains identified by NCBI BLAST. Most RR proteins belonged to the CreB subfamily (Table 2) except for three strains:* L. mirabilis* with an RR of the BasR subfamily (PRK10643) and* A. xylosoxidans* NH44784-1996 plus* A. insuavis* AXX-A with RRs of the QseB subfamily (PRK10336). The second component, PBP, includes two domains LysM and FecR in most cases. Characteristics of the* L. mirabilis* and* P. aeruginosa* PBP components were discussed earlier in Section 3.6. The third component, HK, has an extracellular sensory CHASE2 domain (pfam05226) in all strains. However, the cytoplasmic domains are variable. The second sensor domain, PAS9, was replaced by PAS4 in six strains from different phylogenetic lineages and absent in* L. mirabilis* and in* A. faecalis*. The next two domains, forming multidomain of NtrB subfamily (COG3852) in BiofilmReg operon HK of LBP strain, belong in most cases to the BaeS subfamily (COG0642) even in genus* Burkholderia* (Table 2).

**Figure 4:**
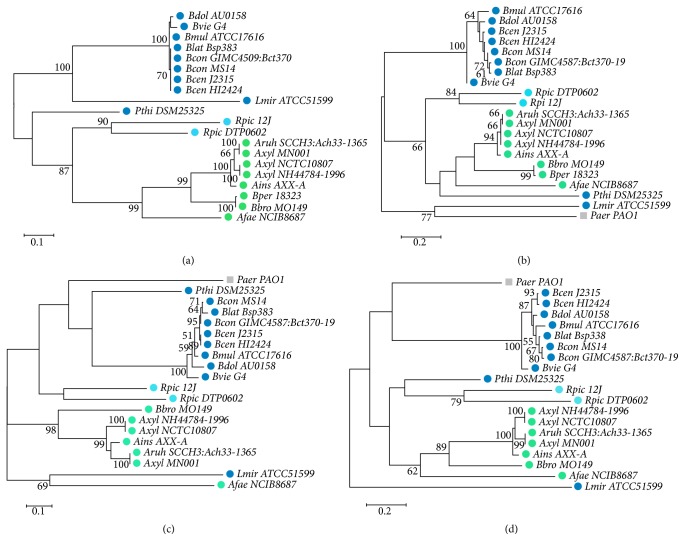
ML phylogenetic trees based on aligned amino acid characters of (a) response regulator (REC signal receiver domain and trans_reg_C effector domain), (b) uncharacterized conserved protein, containing LysM and FecR domains, (c) histidine kinase PAS + HisKA + HATPase_c domains, and (d) histidine kinase CHASE2 domain. Dark blue: Burkholderiaceae, blue: Ralstoniaceae, green: Alcaligenaceae, and grey:* Pseudomonas aeruginosa.* 228 aligned characters of response regulator (REC signal receiver domain + trans_reg_C effector) (a), 213 and 199 aligned characters of histidine kinase (PAS + HisKA + HATPase_c and CHASE2, resp.) (b, c), and 113 aligned characters of peptidoglycan-binding protein (LysM and FecR domain) (d) were taken for phylogeny reconstruction.

The second level of proteins comparison consisted of evaluating phylogenetic relatedness for individual operon components based on the most conservative protein regions. Four resulting alignments are shown in Figure 4. The four resulting ML phylogenetic trees had slightly different topologies.

In general, the trees included two clusters: a* Burkholderia* species cluster and an* Achromobacter* +* Bordetella* cluster. The positions of* P. aeruginosa*,* L. mirabilis*,* A. faecalis, R. pickettii*, and* P*.* thiooxydans* were variable and sometimes unconventional. For instance,* R. pickettii* strains and* P. thiooxydans* DSM 25325 were not merged with Burkholderiaceae species, in contrast to the more generally accepted phylogeny of these taxa. Most RRs exhibited greater similarity than HKs: 38.2% to 100% compared with 31.8–100% and 24.9–100% percent similarity for PAS + HisKA + HATPase_c and CHASE2 domains of HKs, respectively. It can be elucidated by variable specificity of the HK sensor domains.

The* Burkholderia* genus was more representative in our analysis, so we compared proteins similarities inside this genus alone. Most variable among them were the CHASE domains of HK (differences: 1.2–13%), PBP (3.7–24.1%), and UnP (2.4–23.4%) sequences. All these protein domains are localized in the periplasm and have first contacted with signal molecules. Five strains (Burkholderia sp. 383, MS14, G4, HI2424, and ATCC 17616) in our cohort were environment from different ecological niches and three strains (GIMC4509:Bct370, AU0158, and J2315) were host-associated: CF or non-CF patients. We suggest that the variability sequences as revealed here may reflect special adaptive characteristics of the* Burkholderia* strains.

### 3.8. Horizontal Gene Transfer versus Foreign DNA Contamination?

The* Burkholderia* species themselves and other Burkholderiales representatives are the primary soil-dwelling bacteria successfully specialized to different ecological niche, including host-associations. The presence of* P. aeruginosa* has been observed in all these niches, which could explain the acquisition of the BiofilmReg operon by an ancestral* P. aeruginosa* strain. Moreover,* Burkholderia*,* Achromobacter*,* Ralstonia,* and* Pseudomonas* species were previously included together in one genus,* Pseudomonas*. Only the advent of molecular-genetic methods allowed microbiological systematics to split this huge assemblage.

Surprisingly, we found an orthologs of full-size BiofilmReg operon in single genome from Gram-positive bacterium, the actinobacterium* Mumia flava* strain MUSC 201. This is a new genus in the family Nocardioidaceae, which was first approved in 2014 [47]. This strain was originally isolated from mangrove soil in Malaysia. Because horizontal gene transfer (HGT) is a well-known contributor to gene exchange between bacteria, Archaea, and Eukarya, we considered that this might be an example of this process. Orthologs of the BiofilmReg operon have been found in contig 65 of whole shotgun genome* Mumia flava* MUSC 201 (JTDJ01000001–JTDJ01000923) with similarity for RR, PBP, HK, and UnP genes 100.0%, 95.1%, 97.0%, and 96.6%, respectively. However, some observations were highly enigmatic. For example, the* Mumia flava* genome was unexpectedly big: 16.4 Mb, in contrast to the few other Nocardioidaceae genomes that have been assembled into chromosomes, with sizes from 3.1 to 7.6 Mb. Second, contig 65 was very similar in sequence to the reference* B. lata* genome, not only within the borders of the operon but along a 180 kb stretch. Third, in* M. flavia* contigs 1, 12, 134, 150, and 229 we found sequences similar to those of* Burkholderia cenocepacia* J2315,* Burkholderia contaminans* MS14, and* Ralstonia pickettii* 12D, including their* 16S rDNA* sequences. So we obtained the evidence for foreign DNA pollution in* M. flava* strain MUSC 201 genome and made sure exclusively chromosome assembled genomes are verified material for gene analysis.

This means only some Gram-negative bacteria have orthologs of BiofilmReg operon.

## 4. Conclusion

The four-component operon of* Burkholderia contaminans* strain GIMC4509:Bct370, named BiofilmReg, was intriguing by biofilm switching ability and structure organization. It was shown to be unique with respect to the relative locations RR and HK in its operon. Exact orthologs of this operon were found only in the Burkholderiales order of Gram-negative bacteria and not in two* Pseudomonas* strains. Phylogenetic analysis base of* 16S rDNA* gene sequences and in accordance with the operon structure demonstrated the evidence of three-component operon inherence from an ancestral bacterium. During evolution, one lineage acquired a fourth gene and others lost the third component. Mutations, especially in sensor domains, helped to increase biodiversity and allow for adaptation to various ecological niches. So now we can observe* Burkholderia*,* Achromobacter*, and* Ralstonia* species as emerging pathogens. This is a result of shift from living free in a natural habitant to adoption of a host-associated pathogen lifestyle [48]. Multiple antibiotic resistance and biofilm formation help these strains avoid therapeutic drugs. Because* Burkholderia* and* Achromobacter* strains from different species all demonstrated a similar operon structure, there is an opportunity to develop a common drug for all these causative agents.

## Supplementary Material

Table S1: Characteristics of two-component transcriptional regulators (TCTR) genes in reference B. lata genome (Accession Number NC_007510.1).
Table S2: Diversity of two-component transcriptional regulators' domains.

## Figures and Tables

**Table 1:** Representatives of Betaproteobacteria and Gammaproteobacteria, which were checked for presence of at least two adjacent components of operon.

Class	Present operon components	Order/family	Absent operon components
Betaproteobacteria	*Burkholderia contaminans* GIMC4509:Bct370, *Burkholderia lata* str. *Burkholderia *sp. 383, *Burkholderia contaminans* MS14, *Burkholderia dolosa* AU0158, *Burkholderia multivorans* ATCC 17616, *Burkholderia vietnamiensis* G4, *Burkholderia cenocepacia* J2315, HI2424, *Lautropia mirabilis* ATCC 51599, *Pandoraea thiooxydans* DSM 25325	Burkholderiales Burkholderiaceae	*Pandoraea pnomenusa* 3 kgm
	*Alcaligenes faecalis *subsp.* faecalis* NCIB 8687, *Achromobacter xylosoxidans* NH44784-1996, NCTC10807, MN001, *Achromobacter ruhlandii* SCCH3:Ach s33-1365, ST36, *Achromobacter insuavis* AXX-A *Bordetella bronchiseptica* MO149, *Bordetella pertussis* 18323	Burkholderiales Alcaligenaceae	—
	*Ralstonia pickettii* 12J, *Ralstonia pickettii* DTP0602	Burkholderiales Ralstoniaceae	—
	—	Burkholderiales Comamonadaceae	*Variovorax paradoxus* S110, *Acidovorax avenae avenae* ATCC 19860
	—	Burkholderiales Sutterellaceae	*Sutterella parvirubra* YIT
	—	Neisseriales	*Neisseria meningitidis* WUE 2594

Gammaproteobacteria	*Pseudomonas aeruginosa* PAO1, *Pseudomonas fluorescens* PCL1751	Pseudomonadales	*Acinetobacter haemolyticus* ATCC 19194, *Pseudomonas fluorescens* A506, SBW25, BRIP34879, F113, Pf0-1, BBc6R8
	—	Enterobacteriales	*Yersinia intermedia* ATCC 29909, *Yersinia pestis* KIM D27, KIM10+, *Escherichia coli* K-12 substr. MG1655, *Shigella flexneri* 2a str. 2457T, 2a str. 301, *Salmonella enterica enterica* serovar Dublin str. CT_02021853
	—	Xanthomonadales	*Xanthomonas campestris *pv.* campestris* str. ATCC 33913
	—	Legionellales	*Legionella pneumophila pneumophila* Hextuple_2q, Hextuple_3a, Thunder Bay
	—	Vibrionales	*Vibrio cholerae* CP1030(3)

**Table 2:** Main domain of the proteins, coding by BiofilmReg operon genes.

N	Operon gene locus tag/protein domains name				**Strain name** (GenBank: Accession Number)	Operons schematic presentation
	1	2	3	4		
1	1126…1830 (KP288492.1)/ **PRK11083** (**CreB**)	1827…3188 (KP288492.1)/ **LysM**, **FecR**	1…2388 (KU252679)/ **PAS9**, **NtrB**	2426…3040 (KU252679)/ **DUF4136**	***Burkholderia contaminans *strain GIMC4509:Bct370 **(KP288492.1; KU252679)	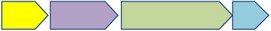
2	BCEP18194_RS13000/ **PRK11083** (**CreB**)	BCEP18194_RS13005/ **LysM**, **FecR**	BCEP18194_RS13010/ **PAS9**, **NtrB**	BCEP18194_RS13015/ **DUF4136**	***Burkholderia lata* strain *Burkholderia *sp. 383 chromosome 1**, **complete sequence **(NC_007510.1)	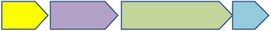
3	NL30_RS08520/ **PRK11083** (**CreB**)	NL30_RS08515/ **LysM**, **FecR**	NL30_RS08510/ **PAS9**, **NtrB**	NL30_RS08505/ **DUF4136**	***Burkholderia contaminans* strain MS14 chromosome 1**, **complete sequence** (NZ_CP009743)	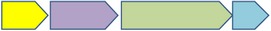
4	AK34_RS21645/ **PRK11083** (**CreB**)	AK34_RS21640/ **LysM**, **FecR**	AK34_RS21635/ **PAS9**, B aeS	AK34_RS21630/ **DUF4136**	***Burkholderia dolosa* AU0158 chromosome 1**, **complete sequence **(NZ_CP009795)	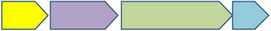
5	BMULJ_RS06770/ **PRK11083** (**CreB**)	BMULJ_RS06775/ **LysM**, **FecR**	BMULJ_RS06780/ **PAS9**, BaeS	BMULJ_RS06785/ **DUF4136**	***Burkholderia multivorans* ATCC 17616 DNA**, **complete genome**, **chromosome 1** (NC_010804.1)	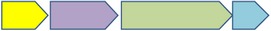
6	BCEP1808_RS07040/ **PRK11083** (**CreB**)	BCEP1808_RS07045/ **LysM**, **FecR**	BCEP1808_RS07050/ **PAS9**, **NtrB**	BCEP1808_RS07055/ **DUF4136**	***Burkholderia vietnamiensis* G4 chromosome 1**, **complete sequence** (NC_009256.1)	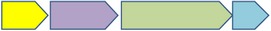
7	QU43_RS43720/ **PRK11083** (**CreB**)	QU43_RS43725/ **LysM**, **FecR**	QU43_RS437230/ **PAS9**, **NtrB**	QU43_RS43735/ **DUF4136**	***Burkholderia cenocepacia* J2315 chromosome 1**, **complete genome** (NC_011000.1)	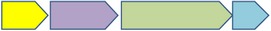
8	BCEN2424_RS07115/ **PRK11083** (**CreB**)	BCEN2424_RS07120/ **LysM**, **FecR**	BCEN2424_RS07125/ **PAS9**, **NtrB**	BCEN2424_RS07130/ **DUF4136**	***Burkholderia cenocepacia* HI2424 chromosome 1**, **complete sequence** (NC_008542.1)	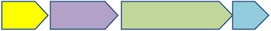
9	HMPREF0551_RS12390/ PRK10643 ( BasR )	HMPREF0551_RS12385/ **LysM**, **FecR**	HMPREF0551_RS12380/ PAS absent, BaeS	HMPREF0551_RS12375/ glutamyl-tRNA reductase	***Lautropia mirabilis* ATCC 51599 genomic scaffold SCAFFOLD1**, **whole genome shotgun sequence** (NZ_GL636062.1)	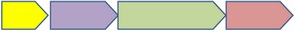
10	QWA_RS04640/ **PRK11083** (**CreB**)	QWA_RS04635/ **LysM**, **FecR**	QWA_RS04630/ PAS absent, BaeS	QWA_RS04625/ AraC family transcriptional regulator	***Alcaligenes faecalis subsp. faecalis* NCIB 8687 Contig_3**, **whole genome shotgun sequence** (NZ_AKMR01000003.1)	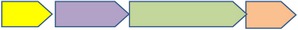
11	NH44784_RS11280/ PRK10336 ( QseB )	NH44784_RS11275/ **LysM**, **FecR**	NH44784_RS11270/ PAS4, BaeS	—	***Achromobacter xylosoxidans* NH44784-1996 complete genome** (NC_021285.1)	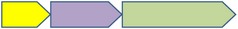
12	ERS451415_06153/ **PRK11083** (**CreB**)	ERS451415_06154/ **LysM**, **FecR**	ERS451415_06155/ **PAS9**, BaeS	—	***Achromobacter xylosoxidans* NCTC10807** (LN831029.1)	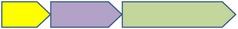
13	Axylo_5268/ **PRK11083** (**CreB**)	Axylo_5269/ **LysM**, **FecR**	Axylo_5270/ **PAS9**, BaeS	—	***Achromobacter xylosoxidans* strain MN001**, **complete genome** (CP012046.1)	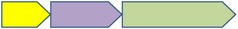
14	Complement (1269…3539)/ **PRK11083** (**CreB**)	Complement (3601…4710)/ **LysM**, **FecR**	Complement (4744…5460)/ **PAS9**, BaeS	—	***Achromobacter ruhlandii* SCCH3:Ach 33-1365**, **ST36** (KU252680)	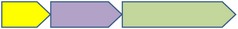
15	AXXA_RS10090/ PRK10336 ( QseB )	AXXA_RS10095/ **LysM**, **FecR**	AXXA_RS10100/ PAS4, BaeS	—	***Achromobacter insuavis* AXX-A genomic scaffold scaffold00003**, **whole genome shotgun sequence** (NZ_GL982453.1)	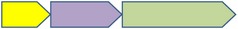
16	BN115_RS00125/ **PRK11083** (**CreB**)	BN115_RS00120/ **LysM**, **FecR**	BN115_RS00115/ **PAS9**, BaeS	—	***Bordetella bronchiseptica* MO149 complete genome** (NC_018829.1)	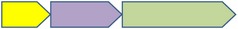
17	BN118_RS00125/ **PRK11083** (**CreB**)	BN118_RS00120/ **LysM**, **FecR**	BN118_RS00115/ **PAS9**, BaeS, no catalitic damain	—	***Bordetella pertussis* 18323 complete genome **(NC_018518.1)	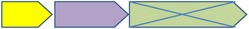
18	RPIC_RS04635/ **PRK11083** (**CreB**)	RPIC_RS04640/ **LysM**, **FecR**	RPIC_RS04645/ PAS4, BaeS	—	***Ralstonia pickettii* 12J chromosome 1**, **complete sequence** (NC_010682.1)	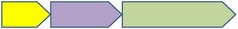
19	N234_RS31485/ **PRK11083** (**CreB**)	N234_RS31480/ **LysM**, **FecR**, **COG4733**, **phage-related protein**, **tail component**	N234_RS31475/ PAS4, BaeS	—	***Ralstonia pickettii* DTP0602 ** chromosome 2, **complete sequence** (NC_022514.1)	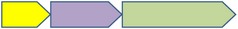
20	—	ABW99_RS09030/ **LysM**, **FecR**	ABW99_RS09035/ PAS4, BaeS	—	***Pandoraea thiooxydans* DSM 25325**, **complete genome** (NZ_CP011568.1)	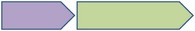
21	—	PA4035/ Lys M truncated, **FecR**	PA4036/ PAS4, BaeS	—	***Pseudomonas aeruginosa* PAO1 chromosome**, **complete genome** (NC_002516.2)	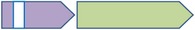

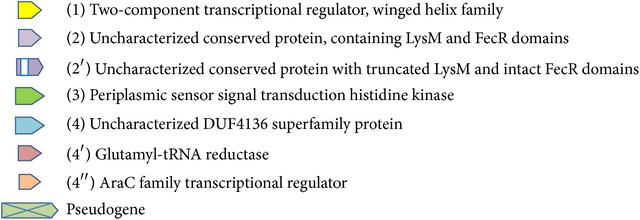

^*∗*^Differences in domain/operon organization or localization are underlined.

## Abbreviations

Bcc: * Burkholderia cepacia* complex
CF: Cystic fibrosis
GIMC: The Gamaleya Institute Microbial Collection
ST: Sequence type
HBP: High biofilm producer
LBP: Lacking biofilm production
NGS: Next generation sequencing
WGS: Whole genome sequencing
NCBI: The National Center for Biotechnology Information
UTR: Untranslated region
CDS: Coding sequence
TCS: Two-component signal transduction system (shortly two-component system)
RR: Response regulator
HK: Histidine kinase
PBP: Peptidoglycan-binding protein
UnP: Uncharacterized protein
ML: Maximum Likelihood
ORF: Open reading frame
BiofilmReg: Biofilm Regulating
TCTR: Two-component transcriptional regulator
wHTH: Winged helix-turn-helix
COG: Clusters of orthologous groups
BI: Bootstrap Index
Blat_B.sp.383: * Burkholderia lata* strain* Burkholderia *sp. 383
Bcon_MS14: * Burkholderia contaminans* strain MS14
Bdol_AU0158: * Burkholderia dolosa* AU0158
Bmul_ATCC17616: * Burkholderia multivorans* ATCC 17616
Bvie_G4: * Burkholderia vietnamiensis* G4
Bcen_J2315: * Burkholderia cenocepacia* J2315
Bcen_HI2424: * Burkholderia cenocepacia* HI2424
Lmir_ATCC51599: * Lautropia mirabilis* ATCC 51599
Afae_NCIB8687: * Alcaligenes faecalis *subsp.* faecalis* NCIB 8687
Axyl_NH44784-1996: * Achromobacter xylosoxidans* NH44784-1996
Axyl_NCTC10807: * Achromobacter xylosoxidans* NCTC10807
Axyl_MN001: * Achromobacter xylosoxidans* MN001
Aruh_SCCH3:Ach33-1365: * Achromobacter ruhlandii* SCCH3:Ach33-1365
Ains_AXX-A: * Achromobacter insuavis* AXX-A
Bbro_MO149: * Bordetella bronchiseptica* MO149
Bper_18323: * Bordetella pertussis* 18323
Rpic_12J: * Ralstonia pickettii* 12J
Rpic_DTP0602: * Ralstonia pickettii* DTP0602
Pthi_DSM25325: * Pandoraea thiooxydans* DSM 25325
Paer_PAO1: * Pseudomonas aeruginosa* PAO1.
